# Activation of Oxidative Stress-Regulated Bcl-3 Suppresses CTCF in Corneal Epithelial Cells

**DOI:** 10.1371/journal.pone.0023984

**Published:** 2011-08-30

**Authors:** Yumei Wang, Luo Lu

**Affiliations:** Department of Medicine, David Geffen School of Medicine, University of California Los Angeles, Torrance, California, United States of America; Louisiana State University and A & M College, United States of America

## Abstract

Epigenetic factor CTCF (CCCTC binding factor) plays important roles in genetic controls of the cell fate. Previous studies found in corneal epithelial cells that CTCF is regulated by epidermal growth factor (EGF) through activation of NF-κB p65/p50. It also found that CTCF is suppressed in ultraviolet (UV) stress-induced corneal epithelial cells. However, it is still unknown how UV stress down-regulates CTCF affecting the cell fate. In the present study, we report that regulation of CTCF by extracellular stress signals is dependent upon activations of an oxidative stress-regulated protein Bcl-3. We found that activated Bcl-3 was able to bind to the κB sites identified in the CTCF promoter region. Bcl-3 was activated by UV irradiation to interact with NF-κB p50 by forming a Bcl-3/p50 heterodimer complex. The Bcl-3/p50 complex suppressed CTCF promoter activity to down-regulate CTCF transcription. Unlike the effect of EGF, UV stress-induced Bcl-3 activation suppressed CTCF activity without involving the IκBα and p65 pathway. Thus, results of the study reveal a novel mechanism for regulatory control of CTCF in UV stress-induced human corneal epithelial cells, which requires activation and formation of Bcl-3/p50 complex through a noncanonical NF-κB pathway.

## Introduction

Corneal epithelial layer plays an important role in the vision function to form the front barrier that defends eye structures behind from damages of physical, chemical and biological insults. Normal wound healing process is particularly important for maintaining the corneal epithelial function [1], [2], [3], [4], [5], [6]. Corneal epithelial wound healing is facilitated by growth factors and delayed by stimulation of environmental stressors [7], [8], [9], [10], [11]. In corneal epithelial cells, growth factors and environmental stressors regulate activities of early response genes and other important transcription factors, including CCCTC binding factor, CTCF. CTCF functions as an epigenetic regulator and transcription factor to control expressions of downstream genes. CTCF is a highly conserved zinc-finger protein and plays multifunctional roles in epigenetic regulations of gene expression, including DNA methylation-sensitive gene insulation, enhancer-promoter blockade, DNA imprinting, and X chromosome inactivation [12], [13], [14]. In corneal epithelial cells, CTCF is significantly activated or suppressed dependent on stimuli to affecting the cell fates [15]. CTCF interacts with multiple genes through recognitions of different DNA targets by variable combinational usage of its 11 zinc fingers within the M domain of CTCF. In epidermal growth factor (EGF)-stimulated cells, CTCF is up-regulated to mediate growth factor-induced proliferation by suppression of *Pax6*, an eye and corneal epithelial specific gene [15]. The recent study demonstrates that EGF stimulation activates NF-κB p65 and p50 subtypes to form heterodimer complex to directly up-regulate CTCF, ultimately leading to corneal epithelial cell proliferation. In contrast, UV stress induces a suppression of CTCF activity in these cells [15]. However, it is still unknown how stresses induce the inhibitory effect on CTCF, which subsequently results in altering corneal epithelial cell fate.

Bcl-3 is an oxidative stress-regulated protein associated with activations of NF-κB p50 and p52 subtypes in the noncanonical NF-κB pathway [16], [17], [18]. Bcl-3 is a member of the ankyrin-repeat-containing IκB family of the NF-κB inhibitors, but it is different from other IκBs. Bcl-3 can be localized in the nuclear compartment in various cell types and is apparently unique containing transactivation domains. Bcl-3 contains two trans-activation domains upstream and downstream from the ankyrin repeats and is able to form dimeric complexes with p50 and p52 subtypes and to regulate gene transcription activities in chronic lymphocytic leukemia cells [19], [20]. In contrast to cytoplasmic IκBs that are degraded in response to many stimulatory signals, Bcl-3 does not undergo regulatory proteolysis. However, the role of Bcl-3 in modulating NF-κB activity has been controversial [21], [22], [23], [24], [25], [26], [27], [28]. It is suggested that overexpression of Bcl-3 can cause dysregulation of genes normally regulated by NF-κB transcription factors to affect cell proliferation, differentiation and apoptosis [20]. In the present study, we demonstrate that Bcl-3 activity was induced by UV stress, but not by EGF stimulation. UV stress-activated Bcl-3 suppressed CTCF by forming heterodimeric complex with active p50. Bcl-3/p50 complex bound to the κB sites identified in the promoter region of CTCF gene. Interaction of Bcl-3 and CTCF gene resulted in suppressing CTCF expression in corneal epithelial cells.

## Results

### Effects of EGF and UV stress on IκBα and NF-κB subtypes

**Figure 1:**
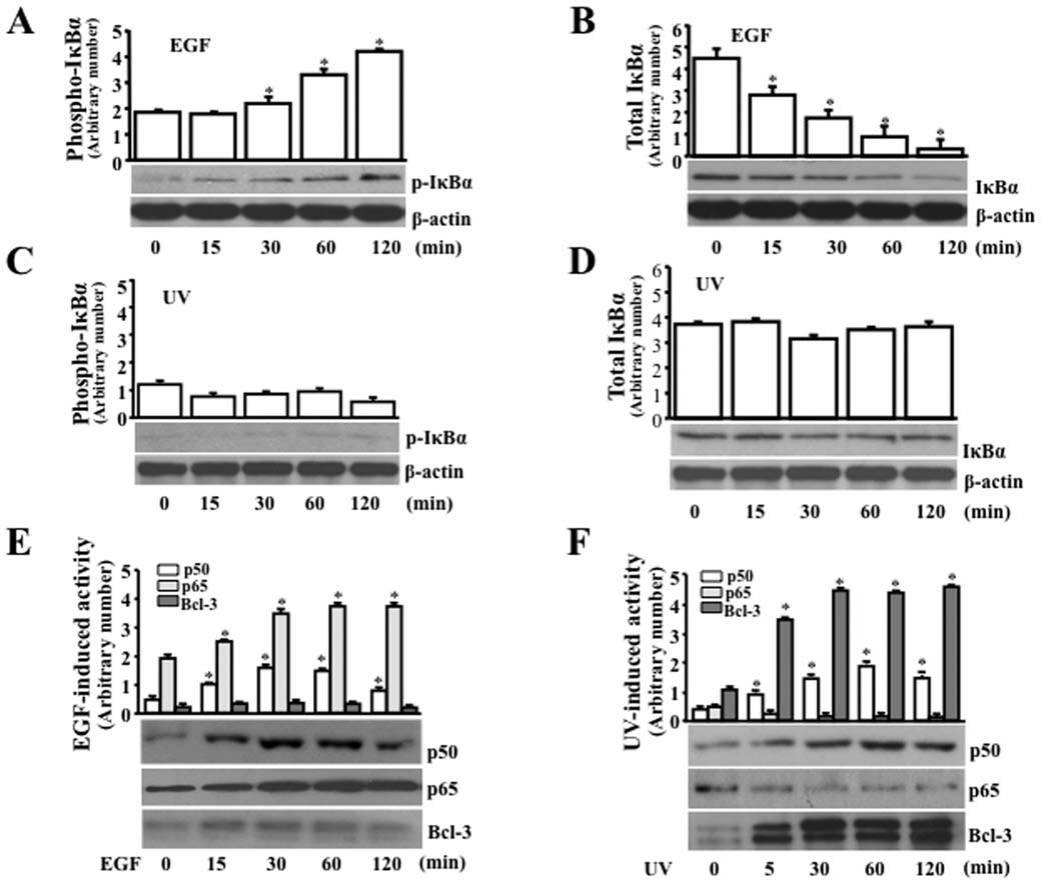
Effects of EGF- and UV stress-induced activation of NF-κB pathways. (A) Time course EGF-induced phosphorylation of IκBα. (B) Time course EGF-induced degradation of IκBα. (C) Effect of UV stress on phosphorylation of IκBα. (D) Effect of UV stress on degradation of IκBα. (E) Effect of EGF stimulation on nuclear activities of p50, p65 and Bcl-3. (F) UV stress-induced nuclear activities of p50, p65 and Bcl-3. HCE cells were synchronized by serum-depletion for 24 h. Total and nuclear proteins were extracted following stimulation at indicated time points and detected by Western analysis. Symbol “*” indicates significant differences between control and induced HCE cells (*p*<0.05, n = 3).

It has shown that phosphorylation of IκBα results in increases in ubiquitination and degradation of the protein, and subsequently releases NF-κB p65 and p50 subtypes that further trans-locate in to the nucleus [29]. We first examined whether EGF and UV stress were able to change the phosphorylation of IκBα. In HCE cells, EGF induced a significant increase in phosphorylation of IκBα following a time course (*Fig. 1A*). EGF-induced phosphorylation of IκBα started at 15 min and reached the peak level at 120 min. There was a marked decrease in total levels of the cellular IκBα in EGF-induced cells from 15 to 120 min tested period, indicating that EGF-induced phosphorylation triggered degradation of the IκBα protein (*Fig. 1B*). In contrast, IκBα phosphorylation was not affected by UV irradiation (*Fig. 1C*). UV irradiation had no effects on the total amount of IκBα either at the indicated time points (*Fig. 1D*). In agreement with the effect of EGF on promoting IκBα phosphorylation and degradation, EGF stimulated p65 and p50 activation, but had no effect on Bcl-3 activity following a time course of 120 min (*Fig. 1E*). In contrast, UV stress stimulated Bcl-3 and p50 activation without activating either IκBα or p60 following a time course of 120 min (*Fig. 1F*). The Results indicate that there are different in effects of EGF and UV irradiation on activation of IκBα and Bcl-3 in growth factor and stress stimulated cells, respectively. The results also indicate that EGF stimulation increased both p65 and p50 levels, while UV stress activated p50 and Bcl-3 in stimulated cells.

### Effect of Bcl-3 activation on CTCF expression

**Figure 2:**
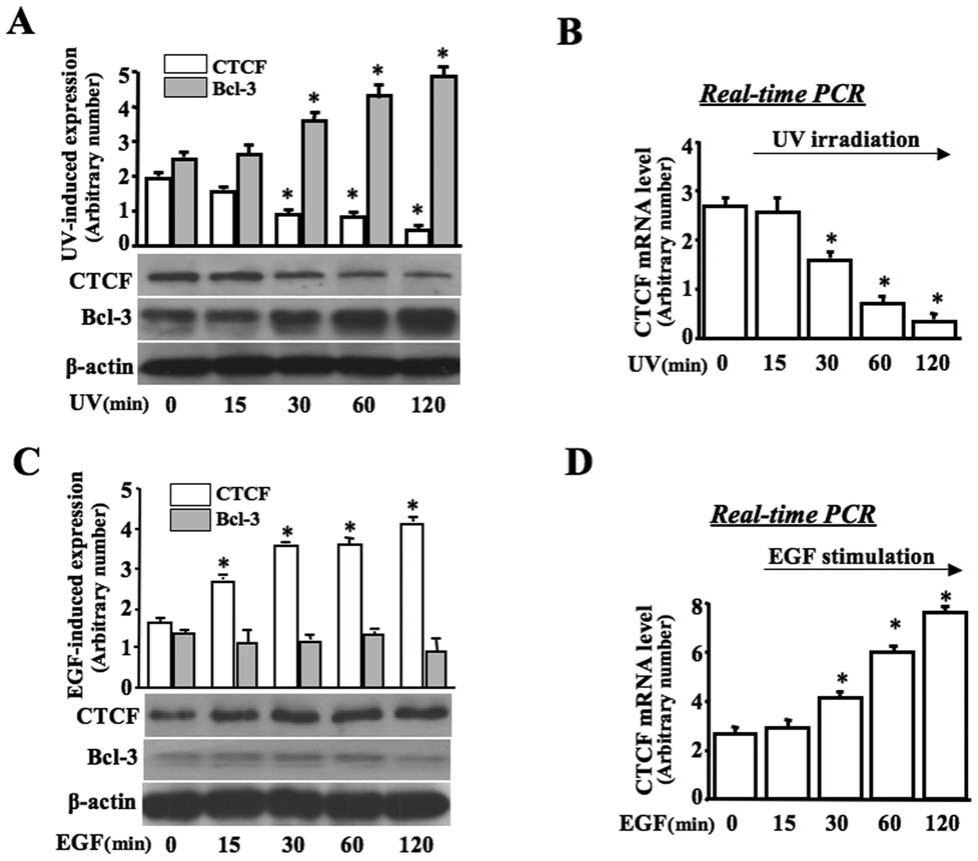
Effect of EGF and UV stress on CTCF activity. (A) Time course of UV stress-induced changes in Bcl-3 and CTCF activity. (B) Quantitative detection of UV stress-induced effect on CTCF mRNA expression by real time PCR. (C) Effect of EGF on Bcl-3 and CTCF activity following a time course. (D) Quantitative detection of EGF-induced effect on CTCF mRNA expression by real time PCR. Proteins and RNA were isolated from HCE cells at indicated time-point before/after EGF (20 ng/ml) and UV irradiation (42 µJ/cm^2^), respectively. Symbol “*” indicates significant differences between control and UV stress-induced cells (*p*<0.05, n = 3).

Previous studies revealed that UV stress induces suppression of CTCF activity in HCE cells. Next question is whether Bcl-3 is an important element that mediates the effect of UV stress on suppression of CTCF. We found that UV stress induced an increase in the Bcl-3 level within the testing period of 30 to 120 min. In the same time, expression of CTCF was significantly suppressed in response to UV irradiation (*Fig. 2A*). Further experiments were done to measure UV stress-induced changes of the CTCF mRNA level. CTCF mRNA expressions were significantly suppressed in UV stress-induced cells measured by reverse-transcription and quantitative real-time PCR (*Fig. 2B*). EGF stimulation induced increases in CTCF expression, but it failed to activate Bcl-3 in EGF-stimulated cells (*Fig. 2C*). In agreement with the Western analysis, CTCF mRNA was also significantly increased in EGF-induced cells measured by quantitative real-time PCR (*Fig. 2D*). Results of measuring UV stress-induced Bcl-3 activation and CTCF suppression suggest that the expression level of CTCF was closely correlated to the altered Bcl-3 activity in UV stress-induced cells.

**Figure 3:**
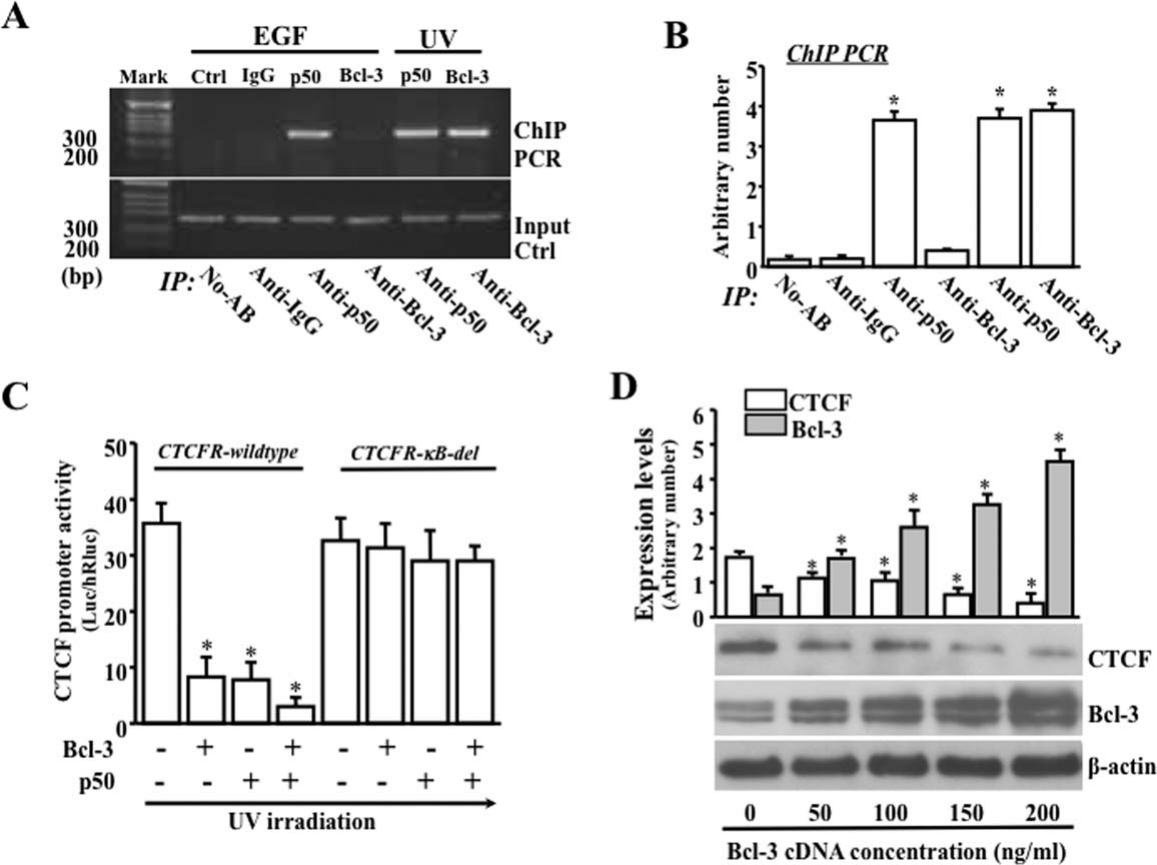
Interaction of Bcl-3 and p50 with CTCF promoter. (A) EGF- and UV stress-induced interactions of Bcl-3 and p50 with CTCF promoter detected by ChIP assays. (B) Analysis of interactions between Bcl-3/p50 and CTCF promoter. (C) Effect of over-expression of Bcl-3 and p50 on activities of wildtype and deletion mutant of CTCF promoter. (D) Dose-response relationship between over-expression of Bcl-3 and suppression of CTCF expression. HCE cells were transfected with full-length cDNAs encoding Bcl-3 (a generous gift from Dr. Shin-Ichiro Takahashi at the University of Tokyo) and p50, CTCF reporter and the mutant CTCF reporter with κB site-deletion by lipofection. Symbol “*” indicates significant differences between control and transfected cells (*p*<0.05, n = 4).

Previous studies indicate that the CTCF promoter region contains NF-κB binding motifs, termed as hCTCFp-κB-sites, which may be responsible for p50 binding to affect CTCF promoter activity [30]. To further characterize the effect of Bcl-3 activation on CTCF expression, we examined whether Bcl-3 can bind to the same κB-site in the region of CTCF promoter by ChIP assays. ChIP analysis showed that Bcl-3 was recruited to the hCTCFp-κB-site. DNA fragments containing the CTCF promoter were pulled down by anti-CTCF antibodies from UV stress-induced cells, but not from EGF-stimulated cells, suggesting that there was a binding site for Bcl-3 to interact with, which is similar to the effect of p50 on the sites of the CTCF promoter region (*Fig. 3A* &* 3B*). Next question was whether specific hCTCFp-κB-site that was observed in p50 binding experiments also requires Bcl-3 binding to carry out its repressor effect. Bcl-3 and p50 were overexpressed in HCE cells that were co-transfected with a CTCF reporter (CTCFR-wildtype) to test for CTCF promoter activity. Overexpression of Bcl-3 resulted in a significant suppression of CTCF promoter activity. However, CTCF promoter activity was not affected in cells transfected with a mutant reporter (CTCFR-κB-del) in that the hCTCFp-κB-site was deleted (*Fig. 3C*). We also examined CTCF expression in Bcl-3-overexpressed cells by Western analysis. It showed that increased concentration of Bcl-3 cDNA in transfected cells resulted in a significantly increased suppression of CTCF (*Fig. 3D*). These results indicate that there was an interaction between Bcl-3 and CTCF promoter and that Bcl-3 suppressed CTCF promoter activity.

### Effect of altered Bcl-3 activity on UV-induced suppression of CTCF

**Figure 4:**
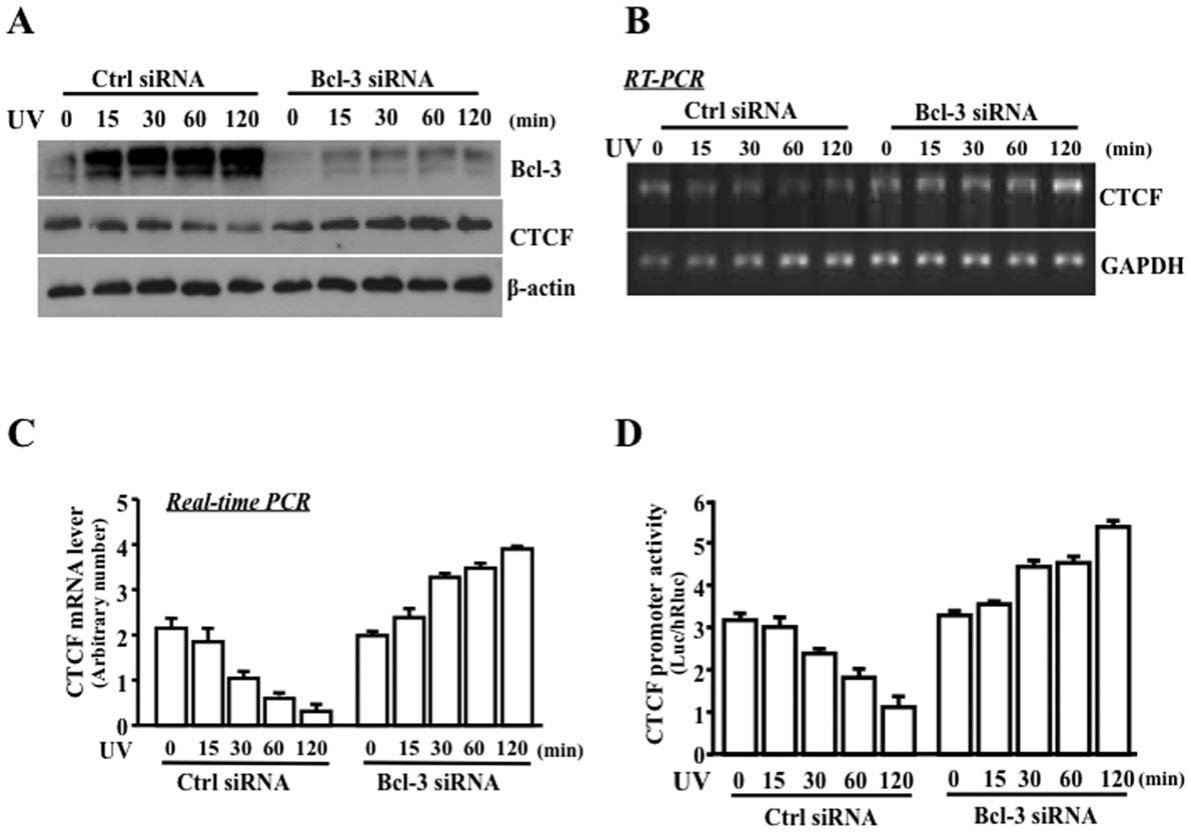
Effects of knocking down Bcl-3 on UV stress-induced suppression of CTCF. (A) Time-dependent effect of knocking down Bcl-3 on UV stress-induced suppression of CTCF detected by Western analysis. (B) Time-dependent effect of knocking down Bcl-3 on UV stress-induced inhibition of CTCF mRNA expression by RT-PCR. (C) Quantitative detection of UV stress-induced CTCF mRNA suppression in Bcl-3 knocking down cells by real time PCR. (D) Effect of knocking down Bcl-3 on UV stress-induced suppression of CTCF promoter activity. Data were plotted as Mean±SE and statistical significance was determined at *p*<0.05 (n = 3 to 6).

As shown above that UV stress-induced Bcl-3 activation down-regulated CTCF, we further verified whether altered Bcl-3 activity has a functional impact on regulation of CTCF in UV stress-induced HCE cells. Bcl-3 was knocked down by transfecting cells with siRNA specific to Bcl-3, and control cells were transfected with non-related siRNA with/without UV irradiation (*Fig. 4A*). The results showed that knockdown of Bcl-3 resulted in increases in CTCF mRNA levels following a time course detected by RT-PCR and by quantitative real-time PCR (*Fig. 4B & 4C*). In addition, knocking down Bcl-3 mRNA abolished the inhibitory effect of UV irradiation on CTCF promoter activity (*Fig. 4D*). The effects of UV irradiation on suppression of CTCF were reversed by silencing Bcl-3 mRNA indicate that Bcl-3 plays an important role in regulating CTCF transcription by control of CTCF promoter activity.

**Figure 5:**
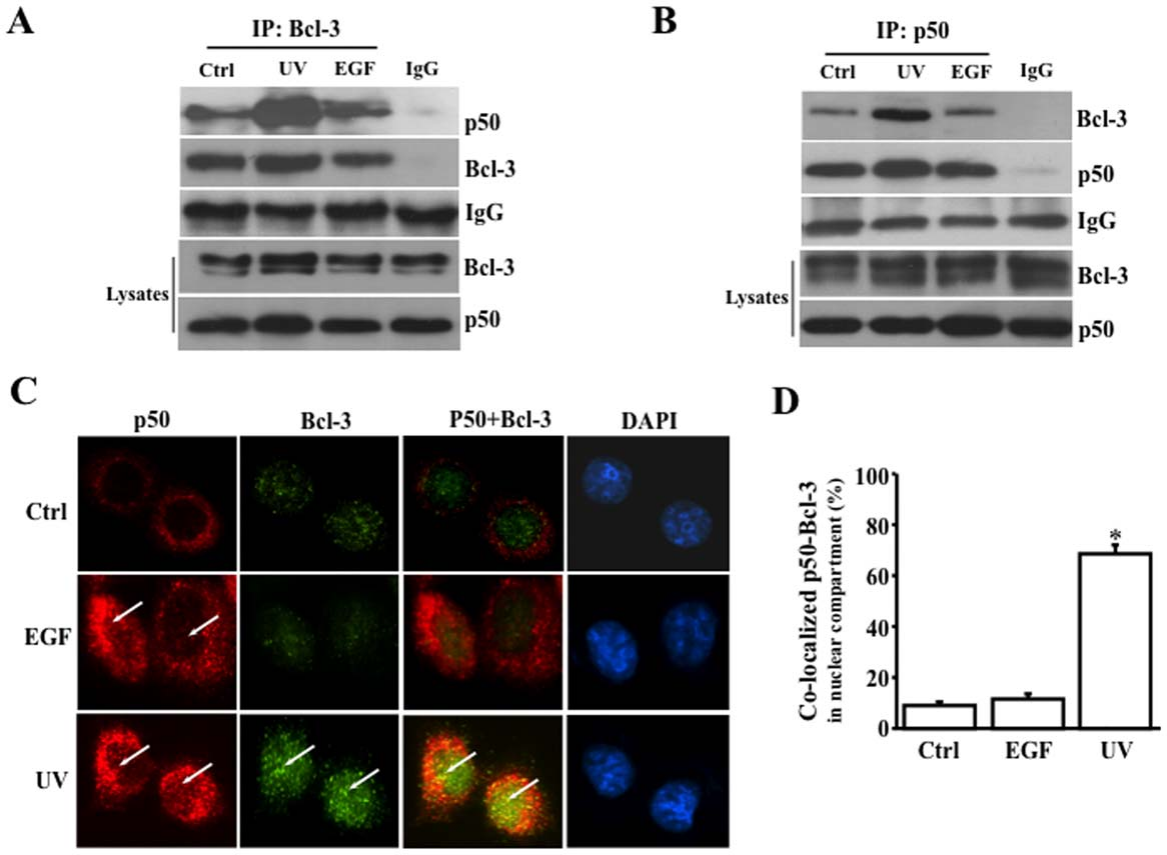
UV stress-induced activation and interaction of Bcl-3 and p50. (A) Immuno-coprecipitation of Bcl-3 and p50 pulled down by anti-Bcl-3 antibodies. (B) Immuno-coprecipitation of Bcl-3 and p50 pulled down by anti-p50 antibodies. (C) Nuclear immuno-colocalization of Bcl-3 and p50 in UV stress-induced HCE cells. Arrows indicate activated p50, Bcl-3 and p50+Bcl-3 localized in the nucleus. (D) Statistical significance of immuno-colocalized nuclear Bcl-3 and p50 in UV stress-induced HCE cells. Bcl-3 and p50 in control, EGF stimulated and UV stress-induced HCE cells were detected by immunostaining experiments with specific antibodies against Bcl-3 and p50. Cell nuclei were detected by DAPI staining. Arrows are indicating immune activities that by were imaged by using a Nikon fluorescent microscope at 40×, and data were analyzed by Nikon software. Symbol “*” indicates significant differences (*p*<0.05, n = 26).

*Interaction between Bcl-3 and p50*. In order to demonstrate the physical interaction between Bcl-3 and p50, Bcl-3 and p50 were pulled down each other by antibodies against Bcl-3 and p50 in EGF- and UV stress-induce cells, respectively. Bcl-3 antibody pulled down more p50 UV stress-induced HCE cell compared with cells stimulated with EGF. No bands were found in the lanes of IgG controls indicating that there was truly a difference of the p50 levels in UV and EGF stimulated cells (*Fig. 5A*). In the meantime, Bcl-3 was specifically immuno-coprecipitated with p50 by anti-p50 antibody in UV stress-induced cells (*Fig. 5B*). However, Bcl-3 did not interact with p50 in EGF-stimulated cells. Total amounts of p50 and Bcl-3 in cell lysates were analyzed by Western blots for input controls. To further investigate whether there is a functional interaction between Bcl-3 and p50, experiments to co-localize Bcl-3 and p50 in nuclei in EGF- and UV stress-induced cells were performed using antibodies labeled with Alexa 488 (green) and 594 (red) against Bcl-3 and p50, respectively (*Fig. 5C*). Bcl-3 and p50 were co-localized in the nucleus that was indicated by DAPI staining in UV stress-induced cells. There was no co-localization of Bcl-3 and p50 found in EGF-induced and un-stimulated cells (control). Further statistical analysis revealed that there was a significant increase in Bcl-3 and p50 co-localization in nuclei of UV stress-induced cells (*Fig. 5D*). Immunoco-localization of Bcl-3 and p50 in the nucleus of UV stress-induced cells suggests that Bcl-3 and p50 are very likely to form a functional heterodimer complex in response to UV stress stimulation. The results provide further evidence indicating that Bcl-3 has functional interactions and physical associations with p50 in the nucleus of UV stress-stimulated HCE cells.

## Discussion

In recent studies, we demonstrate that regulation of CTCF is stimulus-dependent to affect HCE cell fate [15]. Differential stimuli involve activation of NF-κB subtypes that are able to regulate CTCF activity in the NF-κB family. However, there is no detailed information available regarding why UV stress-induced regulation of CTCF is different from the EGF effect since both stimuli all activate the NF-κB pathway. We found previously that EGF-induced activation of p65 and p50 forms heterodimers, which belong to the canonical NF-κB pathway. In the present study, we focus on the effect of UV stress on down regulation of CTCF through activation of a Bcl-3-mediated signaling pathway in HCE cells. In contrast to the EGF effect, we did not observe phosphorylation and degradation of IκBα in UV stress-induced cells, suggesting that there may be an involvement of an alternative NF-κB pathway, such as Bcl-3 that is often seen in oxidative stress-related events demonstrated by previous studies [16].

The present study is for the first time to demonstrate that differential stimuli induce activation of different IκB isoforms in NF-κB pathways, leading to up- and down-regulation of CTCF, respectively. In the canonical NF-κB pathway, EGF significantly promoted a rapid phosphorylation and degradation of IκBα and nuclear translocation of p65, leading to enhancement of CTCF expression following the traditional regulatory mechanism of the NF-κB pathway [31], [32], [33]. In contrast to activation of the canonical NF-κB pathway, UV stress did not induce phosphorylation and degradation of IκBα. Instead, UV stress up-regulated Bcl-3 activity in the nucleus to form Bcl-3/p50 complex that suppressed CTCF expression without involving IκBα and p65, suggesting the involvement of noncanonical NF-κB pathways [34], [35].

We identified that Bcl-3 is involved in UV stress-induced signaling responses linking to CTCF regulation in HCE cells. It has suggested that Bcl-3 is one of the IκB proteins in the noncanonical NF-κB pathway. In the present study, we reveal that UV stress-induced CTCF regulation is very different from what it was observed in EGF-stimulated cells. In consistent to previous published results, phosphorylation and degradation of IκBα in EEGF-induced cells plays a critical role in canonical IκBα-dependent regulation of CTCF transcription. Previous studies also demonstrate that Bcl-3 is associated with p50 or p52 subtypes to form dimeric complex and bound to the promoter region of various genes [36], [37], [38]. The effect of Bcl-3 on inhibition of gene transcriptions may involve enhancing p50 binding to DNA, facilitating p50 nuclear translocation and promoting assembly of p50 dimers [22], [28], [39]. Our results provided further supporting evidence for the effect of forming Bcl-3/p50 complex in UV stress-induced corneal epithelial cells to suppress CTCF promoter activity and expression. Thus, stress-induced CTCF down-regulation by formation of Bcl-3/p50 complex is defined as an event involving the noncanonical NF-κB pathway [16].

We detected Bcl-3 and p50 in NF-κB consensus sites located in the promoter region of CTCF gene (*Fig. 3*). NF-κB dimers bind to κB sites within the promoter region of CTCF is consistent to their actions observed in regulating transcriptions of other target genes [29]. In addition, altered Bcl-3 activity by over-expression of Bcl-3 or by knockdown of Bcl-3 with specific siRNA markedly decreased and increased CTCF promoter activities and CTCF expression, respectively. These results support the notion that Bcl-3 indeed involves regulation of CTCF gene. Bcl-3 activity was significantly enhanced in UV stress-induced cells. This conclusion is supported by observations of nuclear accumulation of Bcl-3, increases in nuclear Bcl-3/p50 complex and interaction of Bcl-3 with κB binding sites located in the promoter region of CTCF gene. Finally, we found that formation of Bcl-3/p50 complex resulted in suppression of CTCF in UV stress-induce HCE cells.

In summary, IκBα and Bcl-3 are two members of the IκB proteins. They function differently in regulating CTCF, which is dependent on stimulation of EGF and UV stress. We found UV stress-induced CTCF regulation is very different from what was observed in EGF-stimulated cells in that phosphorylation and degradation of IκBα plays a critical role in canonical NF-κB-dependent regulation of CTCF transcription. Instead, UV stress-induced Bcl-3 activation directly regulates CTCF transcription activity through a noncanonical mechanism. Most importantly, the present study demonstrates new mechanisms for regulation of CTCF in response to growth factor and UV stress stimulation mediated by important NF-κB IκBα and Bcl-3 mediated pathways, respectively. The new information provided here concern how the environmental stimulation regulates epigenetic factors through various signaling cascades to affect human corneal epithelial cell fates.

## Materials and Methods

### Cell Culture

Human corneal epithelial (HCE) cells (a SV40-immortalized cell line) were cultured in DMEM/F12 medium [11], [40]. The medium contained 10% FBS and 5 ng/ml insulin in a humidified incubator gassed with 5% CO_2_ at 37°C (Invitrogen™ Life Technologies, Grand Island, NY, USA). HCE cells were passed by treatment of 0.05% trypsin–EDTA and seeded with a density of 10^5^/ml. HCE cells were synchronized by serum-deprived culture for 24 h prior to experimental treatments. EGF was applied (20 ng/ml) following various time courses. HCE cells were exposed to UV irradiation (245 nm) at a dosage of 42 µJ/cm^2^.

### Western Analysis

Nuclear proteins were extracted and used in Western blots following a previously described protocol [15]. In brief, HCE cells were rinsed twice with cold PBS and harvested in 0.3 ml lysis buffer containing (mM): 20 Tris/pH 7.5, 137 NaCl, 1.5 MgCl_2_, 2 EDTA, 10 sodium pyrophosphate, 25 β-glycerophosphate, 10% glycerol, 1% Triton X-100, 1 Na-orthovanadate, 1 phenylmethylsulfonyl fluoride, 10 mg/ml aprotinin and 10 mg/ml leupeptin). Lysates were centrifuged at 13,000×g for 15 min at 4°C, and denatured by adding an equal volume of 2× Laemmli buffer and by boiling for 5 min. Each sample containing 20 µg protein was displayed in 8–10% SDS-polyacrylamide gel depended on molecular sizes of target proteins. Proteins in the gel were transferred to polyvinylidene difluoride (PVDF) membranes by a Semi-dry Transfer Cell (Promega). Following blocked with 5% fat free milk in Tris buffered saline with 0.5% Tween-20 (TBS-T) for 1 h at room temperature (RT), the membrane was hybridized with respective primary antibodies at 4°C overnight. Positive protein bands in the PVDF membrane were visualized by using corresponding secondary antibodies and Western Blotting Luminol Reagent kit (Santa Cruz Biotech, Santa Cruz, CA). Primary antibodies used in the experiments included: anti-CTCF (Upstate, 1∶5000 in use), anti-Bcl-3 (Santa Cruz, 1∶1000), anti-p50 (Santa Cruz, 1∶1000), anti-65 (Santa Cruz, 1∶1000), anti-cleaved PARP (Cell Signaling, 1∶1000), anti-β-actin (Sigma, 1∶10000).

### Immunostaining Cytochemistry

HCE cells were grown on glass slides. Cells were rinsed twice with PBS, fixed for 15 min in 4% paraformaldehyde, and then permeabilized with PBS, 0.1% Triton X-100 (PBS-T) for 30 min at room temperature (RT). The cells were blocked by incubation with 10% normal horse serum in PBS-T for 1 h at RT (Jackson ImmunoResearch Labs, PA), followed by double immunostaining with corresponding antibodies. Cells were washed with ice-cold PBS and stained with DAPI. Photos were captured by using a Nikon fluorescent microscope and analyzed by Nikon software programs. EGF- and UV stress-induced interactions were determined by immunoco-precipitation and immunocolalization. Antibodies against Bcl-3, p50 and p65 were individually mixed with protein-A beads on a rotator for 1 h at RT. DSS solution (2 mg disuccinimidyl suberate in 80 µl DMSO) was added to the mixtures and equilibrated for 60 min at RT. The cross-linked mixtures were centrifuged, washed and suspended in 100 µl of binding buffer. After adding 10 µl antibody-beads to lysates, the mixture was gently rotated at 4°C overnight. The mixture was boiled for 5 min and centrifuged to separate precipitated proteins from antibody-beads. Immunoprecipitated products (20 µl each) were used for Western analysis.

### Knockdown of Bcl-3

Double-stranded RNA (dsRNA) nucleotides specifically targeting Bcl-3 were purchased with a sequence of 5′-CAACGTGAACGCGCAAATGTA-3′ (Qiagen, Cat#: SI02654554). Cells were plated in six-well plates and grown to reach 70% confluence. Bcl-3 specific siRNA was transfected using HiPerFect reagent kit following the manufacturer's protocol (Qiagen). Transfected cells were cultured under the normal condition for 48 h before experiments were performed. Control cells were transfected with nonsilencing siRNA using the same protocol.

### Measurements of CTCF Promoter Activity

HCE cells were plated in 24-well dishes and grown to reach 70% confluence. Cells were transfected with indicated plasmids using lipfectamine reagents following the manufacturer's protocol (Invitrogen). Transfected cells were cultured under the normal condition for 48 h before experiments. Extracts were prepared using the Dual Luciferase Assay System (Promega), and luciferase activity was measured by a luminometer (Femtomaster FB by Zylux, Oak Ridge, TN). Promoter activity was analyzed by normalization of luciferase activity with controls.

### Chromatin Immunoprecipitation (ChIP)

Following indicated treatments, HCE cells were fixed for 5 min in 1% formaldehyde and lysed for 10 min in lysis buffer. Chromatin was sheared by sonication to an average size of 1–2 kb and incubated with salmon sperm DNA-saturated protein G-Sepharose beads for 2 h at 4°C. Chromatin mixtures were precipitated overnight at 4°C using 10 µl of antibodies. Immunocomplexes were washed extensively with PBS. Input and immunoprecipitated chromatins were incubated overnight at 65°C to reverse cross-links. After proteinase K digestion, DNA was extracted with phenol/chloroform and precipitated with ethanol. Resulted DNA fragments were dissolved in 20 µl of TE solution. For each sample, 1 µl purified ChIP-DNA or 0.1 µg of the input control DNA was used in PCR reaction. A pair of primers including (5′-TAAGGTCAAGCGGACTGGAT-3′) and the reverse primer- (5′-GGGGGAGGAAAGGTGAGG-3′) located upstream and downstream of the κB site in human CTCF promoter were used in PCR for 25 cycles.

### RT-PCR and Real-time PCR

Total RNA extracted from HCE cells was treated with Trizol reagent (GIBCO-BRL). Reverse transcription was performed using 2 µg of RNA and oligo (dT), and products (4 µl) were used in PCR with a pair of CTCF primers. The forward primer is 5′-CCCTGCGGCTTTTGTCTGTTCTAA-3′ and the reverse primer is 5′-CTGTTTGGGCTGGTTGGTTCTGC-3′. Real-time PCR reactions contained approximately 15–30 ng of cDNA, 5 µl SYBR Green Super mix, and 0.15 µM of each reverse and forward primer specific for those tested genes in 3 sets. Reactions were run for 50 cycles (95°C for 30 sec, 58°C for 30 sec, 72°C for 30 sec) following 2 min initial step at 50°C and 7 min incubation at 95°C. GAPDH was used as a control reference.

### Statistical Analysis

Western blot and DNA gel signals were scanned digitally and some of the optical densities (OD) were quantified with Image Calculator software. Data were shown as mean values plus/minus standard errors (Mean±SE). Significant differences between the control group and treated groups were determined by One-Way ANOVA and Student's *t* test at *P*<0.05.
